# Genome-Wide Identification, Phylogeny and Expression Profile of Vesicle Fusion Components in *Verticillium dahliae*

**DOI:** 10.1371/journal.pone.0068681

**Published:** 2013-07-17

**Authors:** Xue Yang, Siqi Ben, Yingjiao Sun, Xinlei Fan, Chengming Tian, Yonglin Wang

**Affiliations:** 1 The Key Laboratory for Silviculture and Conservation of Ministry of Education, College of Forestry, Beijing Forestry University, Beijing, China; 2 College of Biological Sciences and Technology, Beijing Forestry University, Beijing, China; Iowa State University, United States of America

## Abstract

Vesicular trafficking plays a crucial role in protein localization and movement, signal transduction, and multiple developmental processes in eukaryotic cells. Vesicle fusion is the final and key step in vesicle-mediated trafficking and mainly relies on SNAREs (soluble *N*-ethylmaleimide-sensitive factor attachment protein receptors), the regulators including SM (Sec1/Munc18) family proteins, Rab GTPases and exocyst subunits. *Verticillium dahliae* is a widespread soil fungus that causes disruptive vascular diseases on a wide range of plants. To date, no genes involved in vesicular fusion process have been identified and characterized in *V. dahliae*. The recent publication of the draft genome sequence of *V. dahliae* allowed us to conduct a genome-wide identification, phylogeny and expression profile of genes encoding vesicular fusion components. Using compared genomics and phylogenetic methods, we identified 44 genes encoding vesicle fusion components in the *V. dahliae* genome. According to the structural features of their encoded proteins, the 44 *V. dahliae* genes were classified into 22 SNAREs (6 Qa-, 4 Qb-, 6 Qc-, 1 Qbc- and 5 R-types), 4 SM family proteins, 10 Rab GTPases and 8 exocyst proteins. Based on phylogeny and motif constitution analysis, orthologs of vesicle fusion component in filamentous fungi were generally clustered together into the same subclasses with well-supported bootstrap values. Analysis of the expression profiles of these genes indicated that many of them are significantly differentially expressed during vegetative growth and microsclerotia formation in *V. dahliae*. The analysis show that many components of vesicle fusion are well conserved in filamentous fungi and indicate that vesicle fusion plays a critical role in microsclerotia formation of smoke tree wilt fungus *V. dahliae*. The genome-wide identification and expression analysis of components involved in vesicle fusion should facilitate research in this gene family and give new insights toward elucidating their functions in growth, development and pathogenesis of *V. dahliae*.

## Introduction

Vesicular trafficking is a critical feature for eukaryotic cells, and represents the movement of cargo among between different cell organelles, and between the cell and surroundings. In eukaryotic cells, it is mediated by distinct exocytic and endocytic routes. Endocytosis is a process by which extracellular material is internalized; exocytosis is the secretion of cell synthesized products outside the cell [1]. Studies in budding yeast (*Saccharomyces cerevisiae*), mammals, and plants have shown that vesicular trafficking plays a crucial role in protein localization and movement, signal transduction, and in multiple developmental processes [1]–[3]. Each vesicle trafficking can be divided into four essential steps that includes vesicle budding, transport, tethering and fusion [2]. The fusion process is essential for subcellular compartmentation and the last step in vesicular trafficking, besides the machinery has been conserved from yeast to humans [4].

Protein transport among intracellular compartments is mediated by carrier vesicles that bud from one organelle and fuse selectively with another [5]. A set of key components contributing to vesicle fusion are SNAREs (soluble *N*-ethylmaleimide-sensitive factor attachment protein receptors), tethering complexes, SM (Sec1/Munc18) family proteins and Rab GTPases [6], [7]. SNAREs, core proteins of the membrane fusion machinery, is a super family of membrane-associated proteins characterized by an approximately 65 amino acid α-helical coiled-coil domain called the SNARE motif [4], [8], [9]. The SNARE proteins that mediate vesicle fusion are well studied [10], [11]. Fusion of a transport vesicle with its target membrane is a fundamental process essential to cellular organization and function of all eukaryotic cells [9], [12]. When efficient membrane fusion occurs, four SNARE motifs bundle together forming a coiled-coils configuration [11]. SNAREs function as mediators of fusion between vesicular and target membranes, they can be classified on the basis of their functional classification or their structural classification. Functional classification divides SNAREs into vesicle-associated and target membrane-associated SNAREs (v- and t-SNAREs, respectively) [8], [10], [13]. SNAREs are largely categorized by the sequence similarity of their SNARE domains and by a conserved glutamine (Q) or arginine (R) in the central layer of the domain [10], [14]. They are organized into four classes based on experimental observations: the Qa-, Qb-, Qc-, and R-SNAREs [10], [15]. Generally, t-SNAREs correspond to Q-SNAREs, and v-SNAREs correspond to R-SNAREs. SNAREs are a large family of proteins in most eukaryotes [8], [14]. There are at least 24 SNARE proteins in the budding yeast *S. cerevisiae* [16], [17], 18 in the intra-erythrocytic parasite *Plasmodium falciparum* [18], 62 in *Arabidopsis thaliana* [19], 20 in *Caenorhabditis elegans* [20], 37 in *Homo sapiens* [21] and more than 21 in filamentous fungi, such as *Aspergillus oryzae* [22].

The formation of fusion competent SNARE complexes are regulated by multiple protein families [23], mainly including SM proteins, Rab GTPases and tethering factors such as the exocyst, and these proteins interact either directly or indirectly with SNAREs [8]. Rabs represent a large subgroup of the small G-protein Ras family and complete the set of conserved components that seem to operate at all intracellular transport steps [4]. Rabs have been suggested to regulate the specificity of membrane-trafficking steps because of their distinct subcellular localization and interaction with SNARE components via C-terminal geranylgeranyl moieties [5], [24], [25]. In eukaryotes, the Rab family is involved in cargo selection, recruitment of molecular motor proteins, and interactions with effectors such as SNAREs. Through guanosine nucleotide-dependent conformational transitions within their G-protein domain, Rab proteins undergo a GTP-GDP cycle that is associated with membrane association (GTP form) and membrane dissociation (GDP form). There are thought to be eight types of Rab proteins that are conserved among the majority of eukaryotes [26]. The first Rab proteins to be identified, Sec4 and Ypt1, there is a total of 11 proteins in the yeast Rab family. In fungi, the Rab family is very stable in size and rages from 8 to 12 proteins in most fungi [27]. However, as an exception in fungi, there are 40 Rabs in *Rhizopus oryzae* [28]. The number of Rabs was also analyzed in plant and animal genomes, i.e. *Arabidopsis* contains 57 potential Rab GTPases that are categorized into eight subfamilies (RabA to RabH) [29], and there are at least 66 Rab proteins in *H. sapiens* [28].

A member of the SM protein family is essential for regulating SNARE proteins and SNARE-mediated membrane fusion by directly binding to SNAREs and preventing their entry into nonproductive or premature SNARE complexes [5], [30]. The SM family is a conserved group of hydrophilic proteins containing a conserved about 600 amino acids that fold into an arch-shaped “clasp” structure [24], [31]. *S. cerevisiae* contains four members of the SM protein family [24], the *Arabidopsis* genome contains six members of the SM protein family [29] and humans have seven SM proteins [8].

In addition to Rabs and SM proteins, tethering factors are also essential for SNARE mediated membrane fusion by triggering the engagement of *trans*-SNARE complexes [10]. The tethering complexes are composed of COG, CORVET, Dsl1, exocyst, GARP/VFT, HOPS/Class C VPS, TRAPPI and TRAPPII. Among them, the exocyst is a scaffolding complex which is attached to vesicular membrane and tethers the vesicle to specific domain of the plasma membrane [32]. The exocyst is a large conserved, eight-protein complex including Sec3, Sec5, Sec6, Sec8, Sec10, Sec15, Exo70 and Exo84 and all of the subunits are essential in yeast [33]. All subunits of the exocyst complex appear to be broadly conserved across different fungal genomes [24]. The exocyst is a highly extended structure that is about 30 nm in length, and its components are concentrated in subdomains of the plasma membrane that represent sites of active fusion [34]. Although the components of the SNARE/exocyst secretory machinery are highly conserved among fungi [35], [36], their functions in growth, development and pathogenesis of plant pathogenic fungi are less clear.

*Verticillium dahliae* Kleb. (Eukaryota, Fungi, Ascomycota), an ubiquitous and important soil borne fungal pathogen, causes vascular wilt disease on over 400 host plants, including important agronomical, horticultural, ornamental and woody plants [37]–[39]. Serious outbreaks of the disease caused almost complete yield losses in cotton, potato and sunﬂower [40]–[42]. In recent years, Verticillium wilt of the smoke-tree, *Cotinus coggygria*, which is one of the most important plant species cultivated in landscape ecology, has been caused by *V. dahliae*, resulting in stunted growth of stems, early senescence of leaves, and severe mortality of trees, with seriously detrimental effects on the red leaf scenery in Beijing [43]. No fungicides or control measures are currently available to treat Verticillium wilt once plants have been infected. Despite the economic and ecological importance of this pathogen, the molecular basis of Verticillium disease and its pathogenesis remain poorly understood.

During colonization of the vascular system, *V. dahliae* secretes a reservoir of phytotoxic metabolites that behave as elicitors, inducing phytoalexin formation. *V. dahliae* also produces phytotoxins, which trigger wilt symptoms and vascular discoloration [44]. Substances secreted by *V. dahliae* comprise high-molecular-weight protein-lipopolysaccharide (PLP) complexes, glycoproteins and plant cell wall degrading enzymes, including pectinases, polysaccharidases, and proteinases, which contribute to the pathogenicity of the fungus [38], [45]. Although a secreted VdNEP protein could induce dehydration and wilting, similar to symptoms caused by a crude preparation of *V. dahliae* elicitors [46], and the VdNLP genes could induce necrotic lesions and triggering defense responses in various plants [47], little is known about the mechanisms underlying secretory transport of these proteins out of fungal cells.

In addition, microsclerotia, melanized, multicellular, long-term survival structures, represent a significant developmental event in the life cycle and disease cycle of *V. dahliae* [48]. Microsclerotia are the primary source of disease inoculum in the field. Microsclerotia produce melanin, a dark pigment formed by the oxidative polymerization of phenolic or indolic compounds, which is deposited in the cell walls and intercellular space [49], [50]. How melanin is secreted during microsclerotia formation is remarkably poorly understood. In addition, SNAREs and related proteins are important in fungal secretion and pathogenicity [51], [52]. Thus, we aim to clarify the SNARE mediated vesicle fusion of vesicle trafficking that governs this pathogen’s secretion and pathogenicity. An insight into these trafficking pathways will be useful in designing novel control strategies.

Fortunately, a draft of the *V. dahliae* genome sequence has been reported recently, and the *Verticillium* genomes encode numerous secreted proteins and are similar to those of other plant pathogenic fungi such as *Fusarium spp*. and *Magnaporthe oryzae* [53]. These results suggest that inspection of the vesicle trafficking components of *V. dahliae* and other plant pathogenic fungi with completely sequenced genomes are feasible and important. Therefore, to investigate the number, phylogeny and expression profiles of genes responsible for the last step of vesicle trafficking pathways, vesicle fusion, we undertook a genome-wide identification of genes encoding SNAREs, Rabs, SM proteins and exocysts in this species and other filamentous fungi such as *Fusarium spp*, *Sclerotinia sclerotiorum*, *M. oryzae* and *A. oryzae*. We analyzed the phylogeny of these genes among fungal species. Finally, we reported the differential expression of the above gene families during hyphae, conidia and microsclerotia formation. These results provide important information on the reservoir of secretory proteins and will help to characterize their roles in the growth, development and pathogenesis of *V. dahliae*.

## Materials and Methods

### Fungal Materials and Culture Conditions

This experiment used *V. dahliae* strain XS11, which is a single-spore culture isolated from a smoke-tree in Fragrant Hills Park, Beijing. Cultures were grown on potato dextrose agar. Hyphae were grown in liquid CM (complete medium) [54], and conidia were harvested from cultures grown in liquid CM, as described by Dobinson et al. [54]. To obtain germinated conidia, conidia were diluted in sterile distilled water to 1×10^6^ spores/mL and inoculated in liquid basal medium (BM) by shaking culture for 12 h at 24°C, at 130 rpm, and collected by filtering through two layers of Miracloth. To prepare microsclerotia, conidia were spread over cellulose membranes overlayed onto BM agar (basal agar medium, 10 g/L glucose, 0.2 g/L sodium nitrate, 0.52 g/L KCl, 0.52 g/L MgSO_4_⋅7H2O, 1.52 g/L KH_2_PO_4_, 3 µM thiamine HCl, 0.1 µM biotin, 15 g/L agar, kindly provided by Prof. Katherine Dobinson, Agriculture and Agri-Food Canada, London, Canada), and grown in the dark at 24°C for 14 days.

### Identification of Genes Encoding Predicted SNAREs, Sec1s, Rabs and Exocyst Component Proteins in *Verticillium* and Several Fungi Genomes

Amino acid sequences of SNARE, SM, Rab and exocyst proteins in *S. cerevisiae* were obtained from the SGD database (http://www.yeastgenome.org/) and respectively used to identify the homologs. With these genes as initial queries, systematic BLAST-searches were performed on the *Verticillium* genome database (http://www.broadinstitute.org/annotation/genome/verticillium_dahliae/Blast.html) (tBLASTN, E value ≤0.01). The amino acid sequences of predicted *V. dahliae* proteins showing similarity to yeast proteins were extracted. These sequences were subjected to Pfam motif analysis (http://pfam.sanger.ac.uk/), PROSITE analysis (http://prosite.expasy.org/), and to the SNARE Database (http://bioinformatics.mpibpc.mpg.de/snare/snareSubmitSequencePage.jsp), SM superfamily database 1.75 (http://supfam.org/SUPERFAMILY/cgi-bin/scop.cgi?sunid=56815), and RabDB (http://www.rabdb.org/browse/rabify/) or Rab Database (http://bioinformatics.mpibpc.mpg.de/rab/rabMainPage.jsp) to verify the presence of domains. All genes of *V. dahliae* were subjected to TMHMM Server v. 2.0 (http://www.cbs.dtu.dk/services/TMHMM/), check the presence of transmembrane regions.

The exon/intron organization of individual genes was illustrated using the *Verticillium* Genome structure display website, and partially confirmed by alignment of the gDNA and cDNA sequences amplified by PCR and by sequencing.

We used yeast and *V. dahliae* genes encoding SNARE, SM, Rab and exocyst proteins to identify their counterparts in other available fungal genomes using tBLASTN: *Fusarium* genomes (http://www.broadinstitute.org/annotation/genome/fusarium_group/MultiHome.html), *S. sclerotiorum* genome (http://www.broadinstitute.org/annotation/genome/sclerotinia_sclerotiorum/MultiHome.html), *M. oryzae* genome (http://www.broadinstitute.org/annotation/genome/magnaporthe_comparative/Blast.html), and *A. oryzae* genome (http://www.broadinstitute.org/annotation/genome/aspergillus_group/MultiHome.html). The identified protein sequences were subjected to the above domain search procedures. Synteny and orthology were analyzed and classified by the Multiple Alignment Tool provided by NCBI (http://blast.ncbi.nlm.nih.gov/Blast.cgi).

### Phylogenetic Analysis

The entire amino acid sequences of all SNARE, SM and Rab proteins were aligned using Clustal X (version 1.83) without masking unreliably aligned positions [55]. The maximum parsimony (MP) phylogenetic tree was constructed using PhyML (v3.6.9) under kimura model [56] with 100 replications of bootstrap analysis. Constructed trees were viewed by Treeview (1.6.6) software.

### Nucleic Acid Isolation

Total genomic DNA was extracted from each isolate by a modified CTAB (cetyl trimethylammonium bromide) method using cultures grown on CM with shaking at 150 rpm [57].

Total RNA was isolated from frozen tissues, such as hyphae (HY), conidia (CO), germinated conidia (GC), microsclerotia formation 60 h (MS1), microsclerotia formation 72 h (MS2), microsclerotia formation 96 h (MS3), and microsclerotia formation 14 d (MS4). The samples were ground to a fine powder, and extracted with TRIzol reagent (15596-026, Life Technologies) using procedures recommended by the supplier. For microsclerotia, cultures were grown as described above. The cellulose membrane was removed from the agar, quick-frozen in liquid nitrogen, ground to a fine powder, and then extracted using the TRIzol reagent. Total RNA was eluted in RNase-free water and checked by agarose electrophoresis.

### Quantitative Real-time RT-PCR

qRT-PCR was carried out using SYBR-green fluorescence in an ABI7500 (Roche Applied Science). Gene-specific primers were designed for genes encoding SNAREs, SM proteins, Rabs and exocyst components of *V. dahliae*, preferentially from 3′ end of the gene using Primer 3 with default parameters (http://frodo.wi.mit.edu/primer3/). Two independent biological replicates and three technical replicates of each biological replicate were made for qRT-PCR analysis in vegetative growth stages and microsclerotia formation. The RNA samples were incubated at 37°C for 30 min with DNase I (RNase-free) (D2210, TaKaRa) to remove DNA contamination before RNA reverse transcription. One microgram of RNA was then reverse transcribed into cDNA using an oligo (dT) primer and Invitrogen’s SuperScript III Reverse Transcriptase (11754-050, Life Technologies), according to the manufacturer’s instructions, and diluted 100-fold with nuclease-free water. Primer specificity was further confirmed by dissociation curve analysis obtained after real-time PCR. The expression of each gene in different RNA samples was normalized using the geometric average of the expression levels of two frequently used housekeeping genes, actin and beta-tubulin [48], [58]. The qRT-PCR analyses were performed using 96 well formats with denaturation at 95°C for 3 min, followed by 40 cycles of denaturation at 95°C for 10 s and annealing/extension at 60°C for 31 s. The expression of genes in hyphae or conidia cDNA was assigned the value of 1.0 to allow comparisons to be made between vegetative growth and microsclerotia formation, respectively. The mRNA levels for each gene in different tissue samples were calculated using the 2^−ΔΔCt^ method. ΔΔCt = ΔCt treated sample – ΔCt hyphae or conidia sample (vegetative growth stages or microsclerotia formation were referred to as treatment). Values are the mean of two biological replicates, each with three technical replicates. Error bars indicate the standard deviation.

### Ethics Statement

No specific permissions were required for field studies. The field studies did not involve endangered or protected species.

## Results

### Identification and Classification of SNARE Proteins in *Verticillium* and Other Fungal Genomes

To identify putative SNAREs and related proteins in *Verticillium* and other fungi, we performed a tBLASTN search against the *Verticillium* genomes using SNARE proteins in *S. cerevisiae* as queries and the resulting sequences were used as secondary queries. By removing the redundant sequences, 22 SNARE proteins were identified in *V. dahliae*. All candidates were manually analyzed using the Pfam program (http://pfam.sanger.ac.uk/) to verify the presence of domains. In addition, we further checked and confirmed SNARE motifs and groups in the SNARE Database (http://bioinformatics.mpibpc.mpg.de/snare/snareSubmitSequencePage.jsp). Finally, we designated *Verticillium* SNARE genes following the nomenclature of their orthologs in *S. cerevisiae*.

Fungal SNAREs have been categorized as either Q- or R-SNAREs, depending on whether they contribute a conserved glutamine or arginine, respectively [17]. The detailed information of SNARE genes in *V. dahliae*, including intron number, transmembrane region and protein feature is listed (Dataset S1 and Table S1). The 22 genes of *V. dahliae* were divided into Q- or R-types, and the Q-types were further divided into four sub-types: Qa, Qb, Qc and Qbc. Six genes (VDAG_10168, VDAG_04590, VDAG_04950, VDAG_00278, VDAG_06050 and VDAG_07498) were categorized into Qa-type, 4 genes (VDAG_04124, VDAG_08270 and VDAG_04359, VDAG_01975 ) were categorized into Qb-type, 6 genes (VDAG_05740, VDAG_05532, VDAG_06787, VDAG_01350, VDAG_01236 and VDAG_03579) were categorized into Qc-type and one gene (VDAG_04327) encoding a 480-amimo acid protein which contains two SNARE domains was classified as mixed Qbc SNARE. Four genes (VDAG_08386, VDAG_08948, VDAG_02648 and VDAG_10121) were categorized into R-type. The SNARE motif structure of SNAREs of *V. dahliae* is confined within a minimum sequence length of 53 residues, except for VdSft1 containing 44 amino acids.

Although the annotated *Verticillium* genomes permitted *in silico* analysis to identify the ORFs encoding putative SNARE proteins, we found that there are some errors of annotation of VdBet1 (VDAG_06787) and VdVam7 (VDAG_03579). Based on comparative genomics analysis, we performed PCR and RT-PCR analysis using gDNA and cDNA templates, respectively, extracted from *V. dahliae* XS1 strain. After confirmation by sequencing, the VdBet1 gene was confirmed to have three introns rather than one intron (Text S1). The ORF of VdBet1 was 513 bp, encoding a protein of 171 aa, which had a SNARE motif (VEGILGKVKILKDMTHAIGDEIRDSSAL AEKMNDTFDSTRLRIRGTMNRMLVM) (Text S1). For VdVam7, according to the previous study [17], the N-termini of Vam7 family proteins harbor a Phox homology (PX) domain, which functions as a phosphoinositide binding module. VdVam7 obviously lacks the PX domain at its N-terminus, and the length of protein sequence is much smaller than that of its orthologs in other fungi, suggesting that there is a gap in the current assembly of the *V. dahliae* genome rendering the gene model of the VdVam7 gene incomplete. Unfortunately, we failed to clone the 5′-end of the VdVam7 gene.

To gain further insights into the evolutionary relationships among of SNARE proteins in fungal plant pathogens, we identified SNARE genes from sister species *Verticillium albo-atrum* (another important soil fungus causing wilt disease), as well as five fungal plant pathogens with available whole genome sequences, such as *Fusarium oxysporum* (a causal agent of wilt diseases), *Fusarium verticillioides* (the causal agent of kernel and ear rot of maize), *Fusarium graminearum* (the causal agent of head blight (scab) of wheat and barley), *S. sclerotiorum* (an omnivorous fungal plant pathogen), *M. oryzae* (rice blast fungus) and *A. oryzae* (a fungus known to have prominent potential for the secretory production). After confirming the presence of SNARE motifs, we identified 22 genes encoding SNARE motif in each genome, and they can be categorized into Q- and R-types (Table 1).

**Table 1:** Summary of SNAREs and related proteins in *Verticillium dahliae* and other fungi.

	*Sc*	*V d*	*Va*	*Fo*	*Fv*	*Fg*	*Ss*	*Mo*	*Ao*
SNAREs^a^	26	22	21	22	22	22	22	22	22
Qa	7	6	6	6	6	6	6	6	6
Qb	4	4	4	4	4	4	4	4	4
Qc	6	6	5	6	6	6	6	6	6
Qbc	2	1	1	1	1	1	1	1	1
R	7	5	5	5	5	5	5	5	5
SM family proteins	4	4	4	4	4	4	4	4	4
Rabs	11	10	10	10	10	11	12	11	10
Exocyst component	8	8	8	8	8	8	8	8	8
Total number	49	44	43	44	44	45	46	45	44

a The Q-SNAREs have been further divided as Qa-, Qb-, Qc- and Qbc-SNAREs.

A complete list of the entire deduced amino acid sequences is available in **Dataset S1**. Fungal genome are following: Sc, *Saccharomyces cerevisiae*; Vd, *Verticillium dahlia*; Va, *Verticillium albo*-*atrum*; Fo, *Fusarium oxysporum*; Fv, *Fusarium verticillioides*; Fg, *Fusarium graminearum*; Ss,*Sclerotinia sclerotiorum*; Mg, *Magnaporthe oryzae*; Ao, *Aspergillus oryzae*.

### Identification of SM and Rab Proteins in *Verticillium* and Other Fungal Genomes

To identify SM family and Rab GTPases in *V. dahliae*, tBLASTN searches were used to obtain the amino acid sequences in the *Verticillium* genomes. A total of four genes in the *V. dahliae* genome were identified as possible members of the SM family (Table S2). However, the 4 putative SM family proteins in *V. dahliae* (VDAG_02581, VDAG_06294, VDAG_10115 and VDAG_04476) have a characteristic Sec1 domain, identifying them as members of the SM family proteins.

In addition, the Rab GTPases of *V. dahliae* was mined in the genome by tBLASTN searching. By removing the redundant sequences, a total of 10 Rab family proteins (VDAG_01942, VDAG_07163, VDAG_00886, VDAG_03931, VDAG_06179, VDAG_01437, VDAG_05807, VDAG_08951, VDAG_06883 and VDAG_10156) were identified, all of which contained a monomeric GTPases domain (Table S3).

Next, genes encoding SM and Rab proteins were identified from the previously mentioned six fungal genomes. After confirming the presence of characteristic domain of each protein family, a complete list of SM and Rab proteins identified in this study was provided in Table 1 and **Dataset S1**. As expected, every fungal genome almost contains one counterpart of each ortholog. However, genes encoding YptA were identified in the seven filamentous fungi but not in yeast.

### Identification of Tethering Factor Exocyst Complexes in *Verticillium* and Other Fungal Genomes

Tethering factors have different combinations and number of subunits with the eight complexes, here we identified one component of tethering complexes, exocyst. Using the genomic tBLASTN search strategy, a total of 8 exocyst complex genes (VDAG_00536, VDAG_08435, VDAG_03094, VDAG_09051, VDAG_04051, VDAG_06597, VDAG_05501 and VDAG_00456) were identified in the *V. dahliae* genome (Table S4). Similarly, exocyst complex-encoding genes were also identified in other fungal genomes. In our analysis, all subunits of exocyst complex were found in the seven fungi.

### Protein Structure and Phylogenetic Analysis

**Figure 1:**
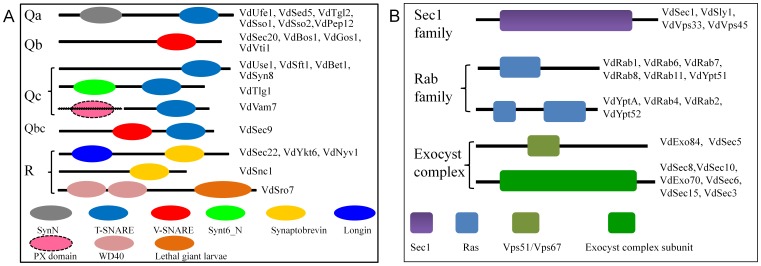
Protein architectures of SNARE, Sec1, Rab and exocyst proteins of *V. dahliae*. Domain structures are drawn to represent their relative positions along the protein chain. Types of proteins are listed on the left, and the protein names are listed on the right. The black solid line represents the corresponding protein and its length. The different-colored boxes represent different domains and their positions in each type of proteins. The domain features were generated by the Pfam program (http://pfam.sanger.ac.uk/). A. a total of 22 SNARE proteins of *V. dahliae*. B. SM, Rab and exocyst proteins of *V. dahliae.* A dotted line and an ellipsoid represent the amino acid sequence and PX domain in the N-terminus of VdVam 7, which is not yet identified because of incomplete sequence.

**Figure 2:**
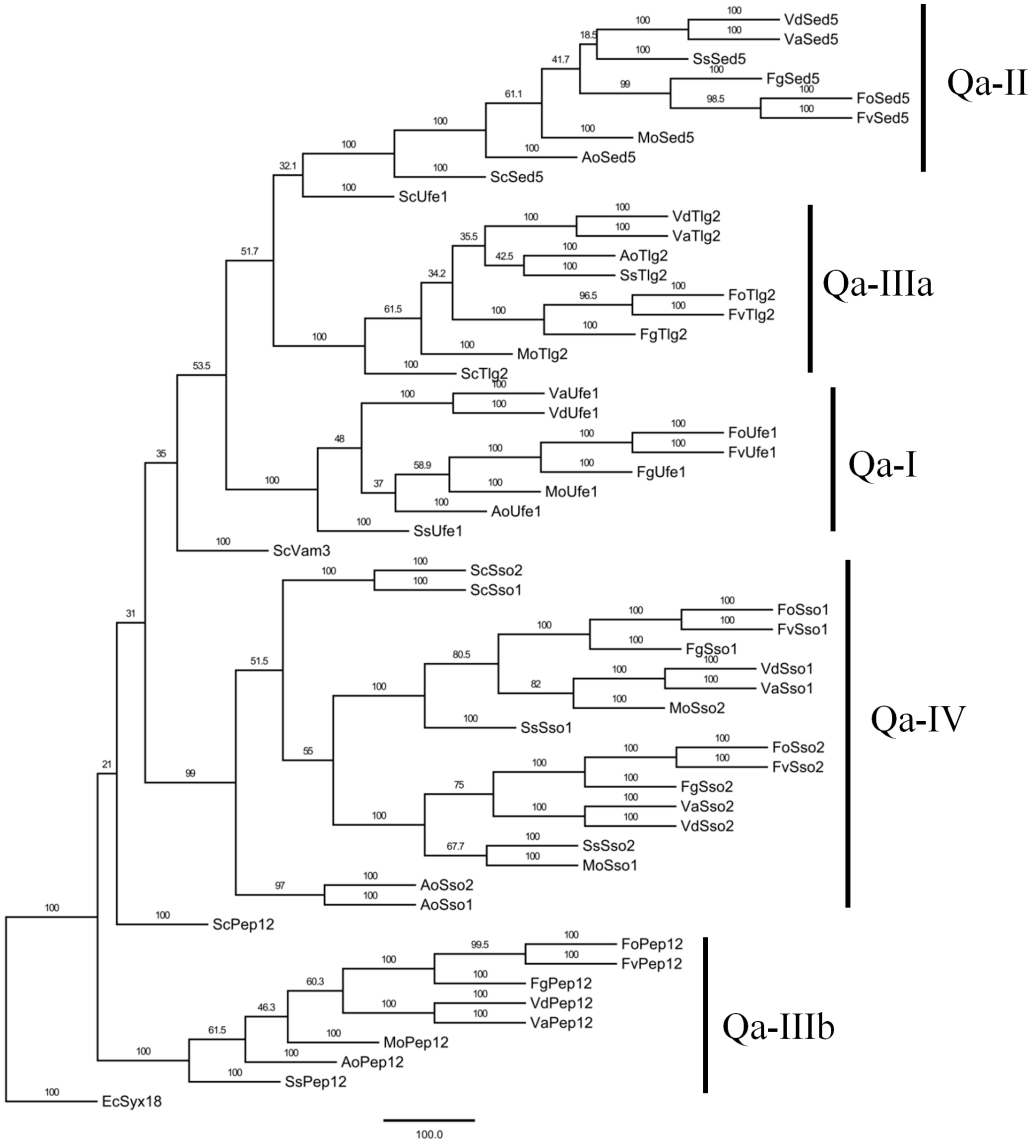
Phylogenetic analysis of Qa-SNAREs from *Verticillium* and other fungal species. The entire amino acid sequences of Qa-SNARE proteins of *Verticillium* and other fungi were aligned using Clustal X and the MP phylogenetic tree was constructed using PhyML package with kimura model amino acid substitution model under 100 replicates of bootstrap analysis. Separate clades are indicated by vertical bars, branch times values were given and the orthologs in different filamentous fungi appeared to cluster together. Qa-type Syx18 of *Encephalitozoon cuniculi* was used as an outgroup. Fungal species are following: Sc, *Saccharomyces cerevisiae*; Vd, *Verticillium dahlia*; Va, *Verticillium albo*-*atrum*; Fo, *Fusarium oxysporum*; Fv, *Fusarium verticillioides*; Fg, *Fusarium graminearum*; Ss, *Sclerotinia sclerotiorum*; Mg, *Magnaporthe oryzae*; Ao, *Aspergillus oryzae*.

To investigate the characteristics of genes encoding SNAREs, SM family, Rab-GTPases and exocyst, the detailed predictions of domain architectures were determined using several bioinformatics websites. We analyzed full-length sequences of SNAREs, SM proteins, Rabs and exocyst of *V. dahliae* to detect the distribution of various domain structures. As displayed schematically in Figure 1, the protein architecture across all the genes of *V. dahliae* were identified and generated in this study. Qa-type SNARE proteins contain SynN (SyntaxinN) domains and a t-SNARE domain (Figure 1A). To unveil the phylogenetic relationships among fungal Qa-SNARES, the MP phylogenetic tree was constructed using the full-length Qa-type SNARE protein sequences of *V. dahliae* and the above eight fungal species by PhyML. Qa-SNAREs of all nine fungal species were classified into five well-conserved subclasses, such as Qa-I, II, IIIa, IIIb and IV (Figure 2). Generally, the orthologs in filamentous fungi appear to cluster together into the same subclasses, but some exceptions were found in yeast genes, suggesting that yeast Qa-SNAREs appear to be lineage-specific and different with filamentous fungi. Yeast Vam3 and Pep12 were not phylogenetically close to other members of Pep12/Vam3 (Figure 2).

**Figure 3:**
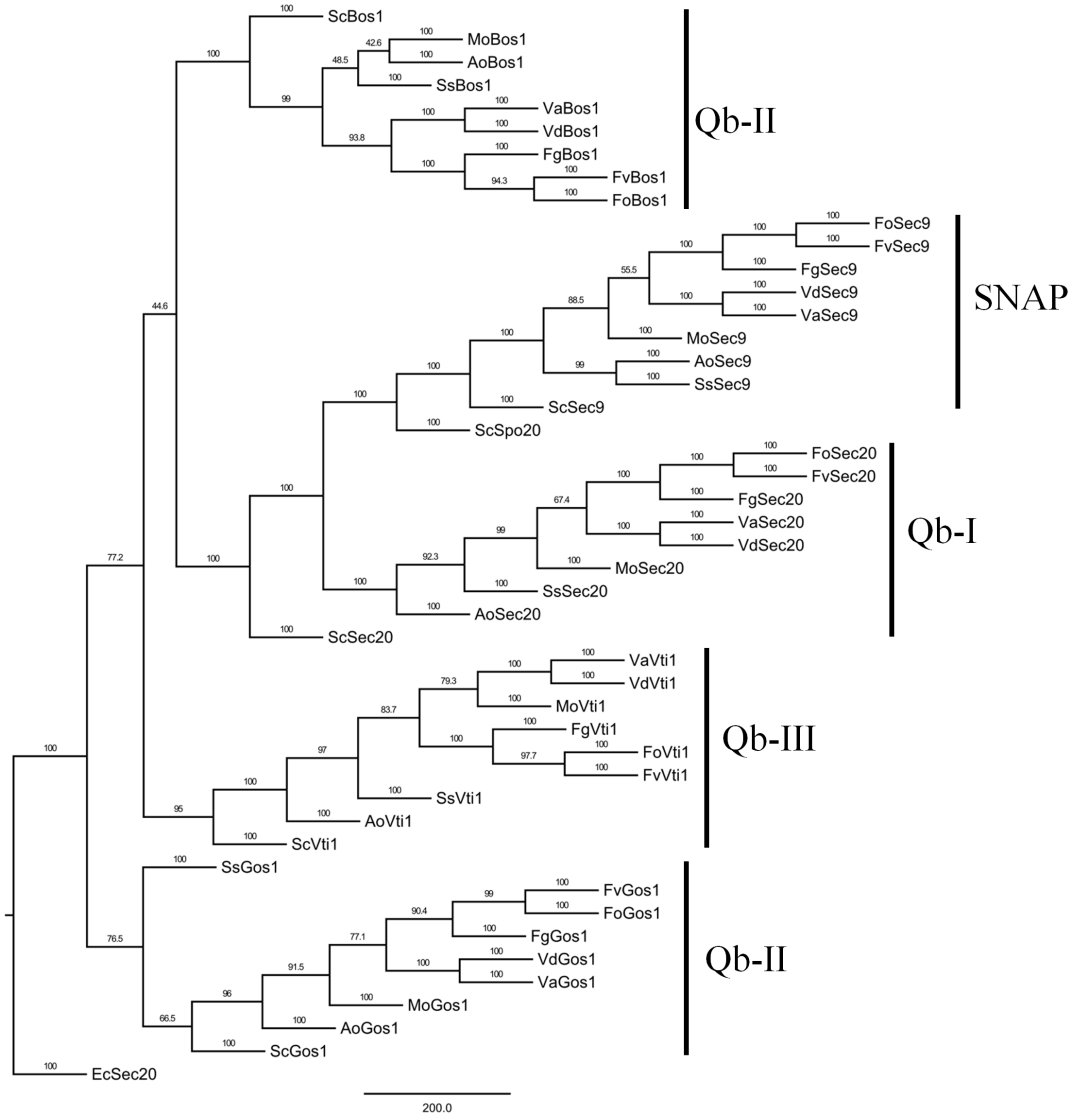
Phylogenetic analysis of Qb-SNAREs from *Verticillium* and other fungal species. The entire amino acid sequences of Qb-SNARE proteins of *Verticillium* and other fungi were aligned using Clustal X and the MP phylogenetic tree was constructed using PhyML package with kimura model under 100 replications of bootstrap analysis. Separate clades are indicated by vertical bars, branch times values were given and the orthologs in different filamentous fungi appeared to cluster together. Qb-type Sec20 of *E. cuniculi* was used as an outgroup.

Qb-type SNAREs have only a V-SNARE and aV-SNARE_C (the latter part of the t_SNARE superfamily) domains. Sec9 is known to contain a tandem of SNARE domain, VaSec9 protein had one of the Qb and one of the Qc type (Figure 1A). A MP phylogenetic tree of Qb-type SNAREs was also constructed by PhyML. In this study, Qb-type SNARE proteins were classified into five clades belonging to five subclasses, such as Qb-I, II, III and SNAP (Figure 3). The phylogenetic tree showed that fungal Sec9 proteins were grouped into in clade SNAP and the tree also revealed that the Qb-SNARE orthologs in fungi clustered together into the same subclasses with well-supported bootstrap values.

**Figure 4:**
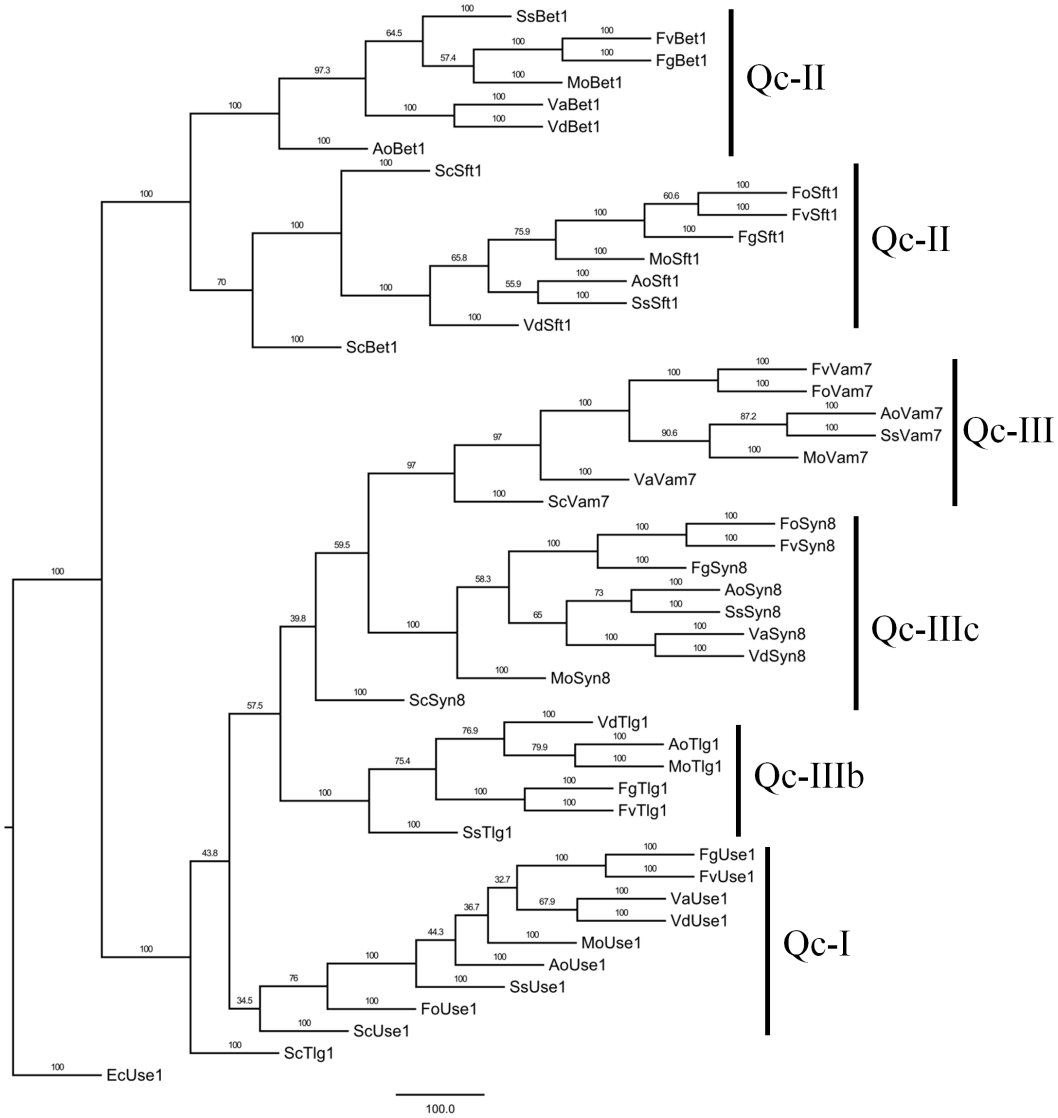
Phylogenetic analysis of Qc-SNAREs from *Verticillium* and other fungal species fungal species. The entire amino acid sequences of Qc-SNARE proteins of *Verticillium* and other fungi were aligned using Clustal X and the MP phylogenetic tree was constructed using PhyML package with kimura model under 100 replicatations of bootstrap analysis. Separate clades are indicated by vertical bars, branch times values were given. Qc-type Use1 of *E. cuniculi* was used as an outgroup.

Qc-type SNAREs have three classes of domain architectures, class 1 has a V-SNARE domain (VdUse1, VdSft1, VdBet1, VdSyn8), class 2 has Syntaxin-6_N and T-SNARE domains (VdTlg1), and class 3 (comprising only VdVam7) harbors a V-SNARE domain and a PX domain as yet unidentified (the PX domain is missing due to incomplete sequence information) (Figure 1A, dotted line and ellipsoid). A MP phylogenetic tree of Qc-type SNAREs was also constructed by PhyML. From the phylogenetic tree, we noted that Qc-type SNARE proteins were classified into six subclasses, such as Qc-I, II, III, IIIb and IIIc (Figure 4). The phylogenetic tree revealed that the Qc-SNAREs in filamentous fungi clustered together into the same subclasses with significant bootstrapping support except two genes of yeast, ScTlg1 and ScBet1.

**Figure 5:**
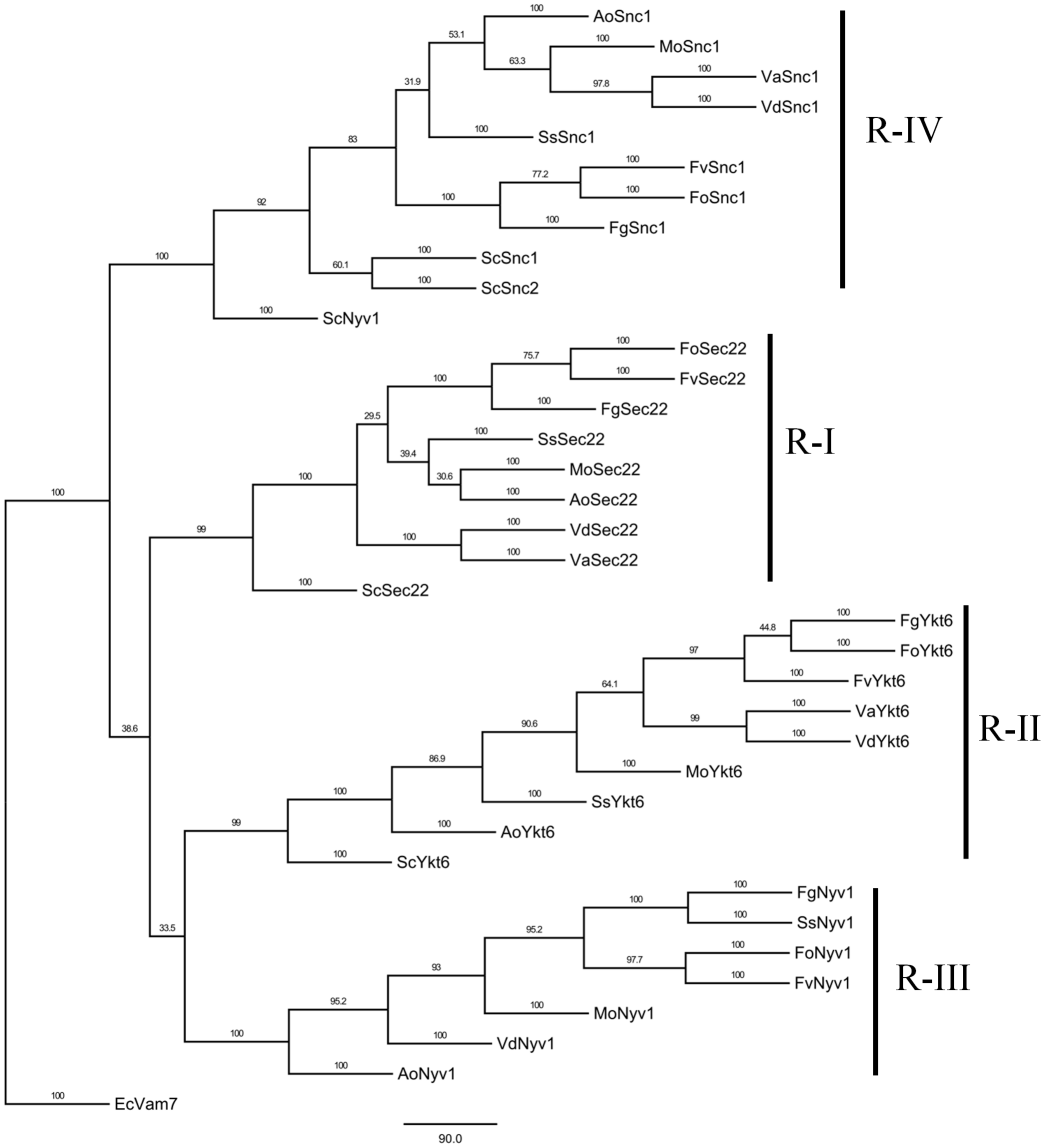
Phylogenetic analysis of R-SNAREs from *Verticillium* and other fungal species. The entire amino acid sequences of R-SNARE proteins of *Verticillium* and other fungi were aligned using Clustal X and the MP phylogenetic tree was constructed using PhyML package with kimura model under 100 replicationss of bootstrap analysis. Separate clades are indicated by vertical bars, branch times values were given. Qc-type Vam7 of *E. cuniculi* was used as an outgroup.

As for R-type SNARE proteins, VdSec22, VdYkt6 and VdNyv1 contain Synaptobrevin and Longin domains, and VdSnc1 has only a Synaptobrevin domain. Sro7 is a novel regulatory SNARE protein in yeast, VdSro7 is a ortholog of Sro7 in *V. dahliae*. VdSro7 contained two WD40 domain sand a lethal giant larvae domain (Figure 1A). A MP phylogenetic tree of R-type SNAREs was also constructed by PhyML. The phylogenetic tree showed that R-type SNARE proteins were classified into four subclasses, such as R-I, II, III and IV (Figure 5). The results also revealed that the R-SNARE orthologs in fungi clustered together into the same subclasses with significantly high bootstrapping support.

**Figure 6:**
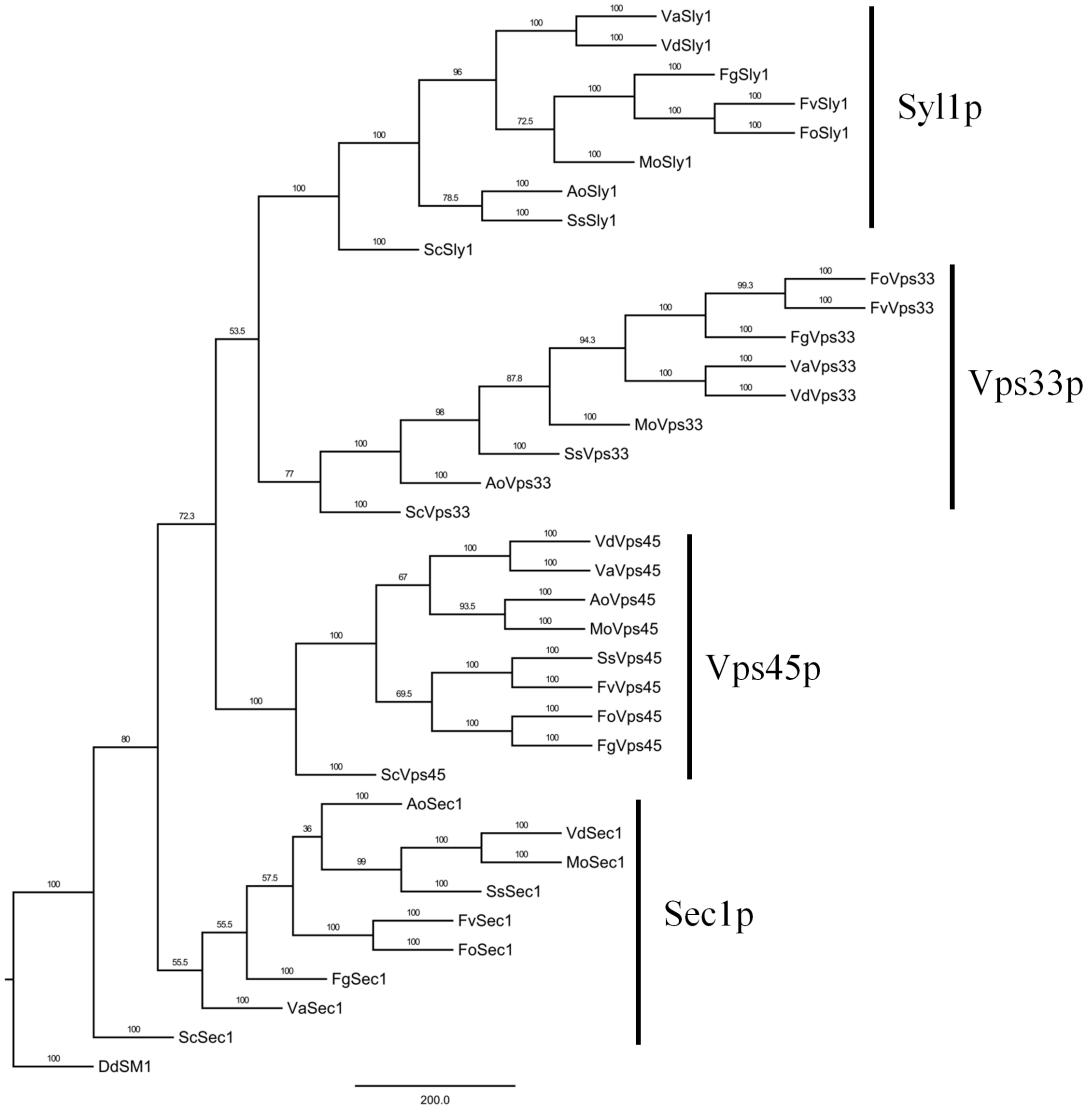
Phylogenetic analysis of SM prteins from *Verticillium* and other fungal species. The entire amino acid sequences of SM proteins of *Verticillium* and other fungi were aligned using Clustal X and the MP phylogenetic tree was constructed using PhyML package with kimura model under 100 replications of bootstrap analysis. Separate clades are indicated by vertical bars, branch times values were given. *Dictyostelium discoideum* Sec1-like (DDB0238598) was used as an outgroup.

The characteristic domain of SM family proteins is the Sec1 domain (Figure 1B). To real the phylogenetic relationship among fungal SM proteins, a MP phylogenetic tree of fungal SM family proteins was constructed by PhyML. As in the SM identification by tBLASTN, there was a very good support for the robust separation of clades representing homologs of Sec1p, Sly1p, Vps33p and Vps45p (Figure 6). The data showed that clades of Syl1p, Vps33p and Vps45p were supported with high bootstrap values, nevertheless, within the clade Sec1, there was a little divergence.

**Figure 7:**
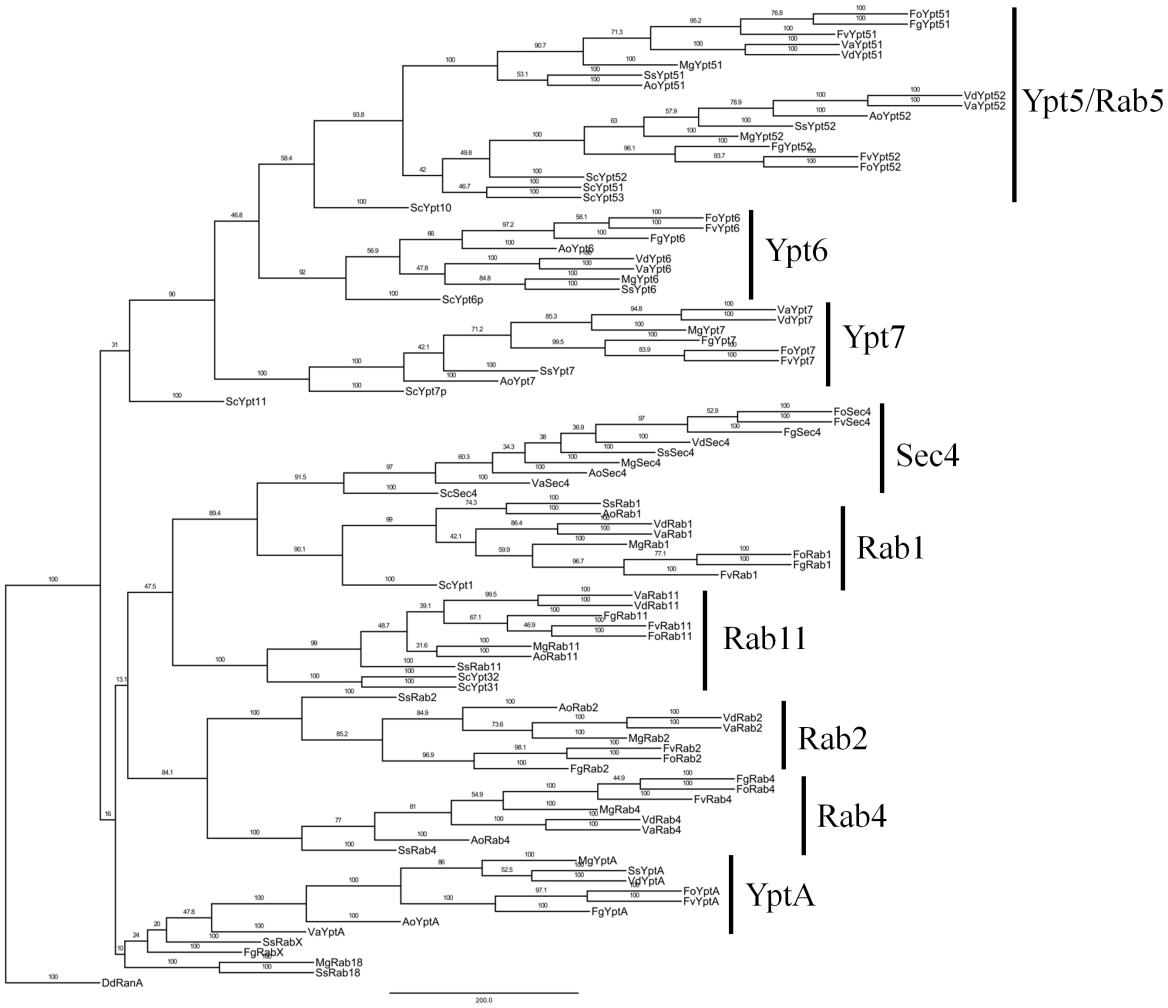
Phylogenetic analysis of Rab GTPases from *Verticillium* and other fungal species. The entire amino acid sequences of Rab-GTPases of *Verticillium* and other fungi were aligned using Clustal X and the MP phylogenetic tree was constructed using PhyML package with kimura model under 100 replications of bootstrap analysis. Separate clades are indicated by vertical bars, branch times values were given. *D. discoideum* RAN (EAL61601) was used as an outgroup.

Rab family proteins have a Ras GTPase domain, six proteins such as VdRab1, VdRab6, VdRab7, VdRab8, VdRab11 and VdYpt51 were identified to have a Ras GTPase domain, the other four proteins such as VdYptA, VdRab4, VdRab2 and VdYpt52 were identified to have two Ras GTPase domains (Figure 1B). To examine the phylogenetic relationships among the fungal Rab family proteins, a MP phylogenetic tree of fungal Rab family proteins was also constructed by PhyML. Consistent with the sequence homology, the phylogenetic tree revealed gene duplication and diversification of the fungal Rab family proteins (Figure 7). The phylogenetic tree revealed that fungal Rabs could be divided into nine large clades (Figure 7). These data showed that well-supported relationships between many Rab paralogs such as Ypt6, Ypt7, Sec4 and Rab1 were reconstructed and major super-clade containing Rab 5 (Ypt51/52/53) was also reconstructed with high support values. Notably, the phylogenetic tree failed to support the relationship between Rabx paralogs, indicating that these Rab-GTPases might be species-specific.

As for the protein structure of exocyst components, VdExo84 and VdSec5 have a Vps51/Vps67 domain; others (VdSec8, VdSec10, VdExo70, VdSec6, VdSec15 and VdSec3) have exocyst complex subunit domains (Figure 1B).

### Expression Analysis of SNARE, SM, Rab and Exocyst Proteins in Vegetative Growth Stages of *V. dahliae*

**Figure 8:**
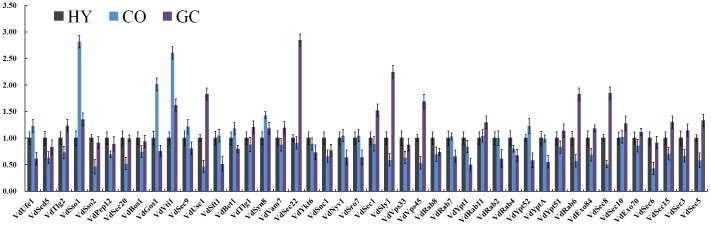
Differential expression profiles of SNARE, SM, Rab and exocyst proteins during vegetative growth of *V. dahliae*. The relative mRNA abundances of genes encoding SNARE, SM, Rabs and exocyst proteins were normalized with respect to reference genes actin and tubulin of *V. dahliae* in hyphae (HY), conidia (CO) and germinated conidia (GC). Error bars represent standard deviations (SD) of three technical replicates. The expression of genes in hyphae was assigned the value of 1.0 to allow comparisons among vegetative growth stages. A. Relative mRNA level of SNAREs. B. Relative mRNA level of SM, Rab and exocyst proteins.

We investigated that expression pattern of SNARE, SM, Rab and exocyst proteins using quantitative real-time RT-PCR (qRT-PCR) methods in the normal vegetative growth stages of *V. dahliae*, including hyphae (HY), conidia (CO) and germinated conidia (GC). All of the predicted genes were expressed in at least one vegetative growth stage (Figure 8). The expression of genes in hyphae was set as the control. Among 44 genes, 18 (40%) genes were significantly differentially (P<0.05) or specially expressed in only one stage or pairs of stages, such as hyphae, conidia and germinated conidia, respectively. Differentially upregulated genes were as follows, VdSso1, VdGos1, VdVti1, VdUse1, VdSec22, VdSec1, VdSly1, VdVps45, VdRab6 and VdSec8). The downregulated genes were VdSso2, VdSec20, VdSnc1, VdSft1, VdYpt52, VdYptA, VdSec6 and VdSec5 (Figure 8).

### Differential Expression Profiles of SNARE, SM, Rab and Exocyst Proteins during Microsclerotia Formation of Smoke Tree Wilt Fungus *V. dahliae*

**Figure 9:**
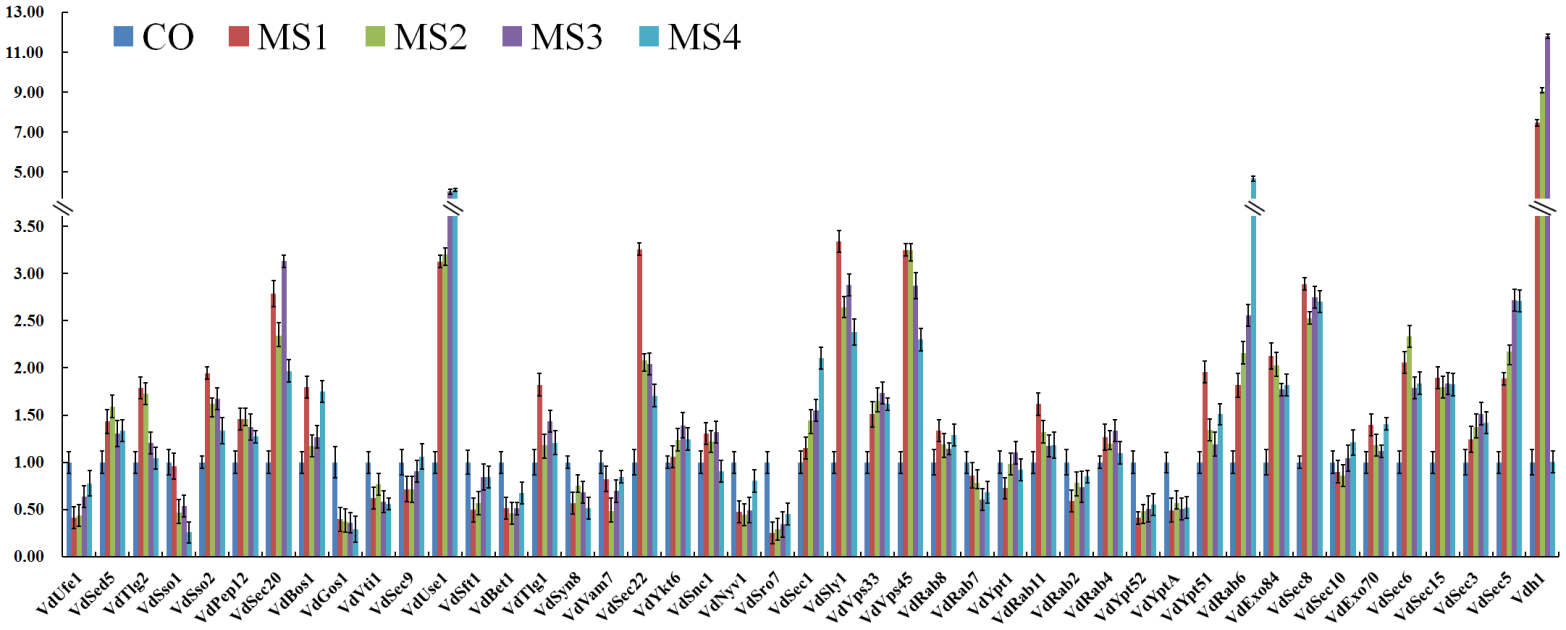
Differential expression profiles of SNARE, SM, Rab and exocyst proteins during microsclerotia formation of *V. dahliae*. The relative mRNA abundances of genes encoding SNARE, SM, Rab and exocyst proteins were normalized with respect to reference genes actin and tubulin of *V. dahliae* in conidia (CO), microsclerotial formation MS1 (60 h), MS2 (72 h), MS3 (96 h) and MS4 (14d). Error bars represent standard deviations (SD) of three technical replicates. The expression of genes in conidia (CO) was assigned the value of 1.0 to allow comparisons among microsclerotia formation stages. A. Relative mRNA level of SNAREs. B. Relative mRNA level of SM, Rab and exocyst proteins.

The formation of melanized, multicellular survival structures referred to as microsclerotia manifests a significant developmental event in the life cycle and disease cycle of *V. dahliae*. In our previous study, microsclerotia formation from conidia clearly represents four stages, such as microsclerotia initiation, middle phase (formation of small clusters of lightly pigmented cells), late phase (formation of large clusters of heavily pigmented cells) and mature microsclerotia (Xiong, et al., unpublished data). To investigate the expression profiles of SNARE, SM, Rab and exocyst proteins during microsclerotia formation, we conducted qRT-PCR assays of these genes plus VDH1, which was confirmed to be involved in microsclerotia development [48]. The expression of VDH1 in this study is similar to that in the previous study of Klimes et al. [48], [59]. Here, the VDH1 gene was significantly upregulated during microsclerotia formation (Figure 9). Among the 44 predicted genes, 30 genes (68%) were significantly (P<0.05) upregulated (19 genes) or downregulated (11 genes) compared with the control (conidia, CO) in at least one stage of microsclerotia formation (Figure 9). Nineteen upregulated genes were: VdSed5, VdTlg2, VdSso2, VdSec20, VdBos1, VdUse1, VdTlg1, VdSec22, VdSec1, VdSly1, VdVps33, VdVps45, VdYpt51, VdRab6, VdExo84, VdSec8, VdSec6, VdSec15 and VdSec5. The 11 downregulated genes were: VdUfe1, VdSso1, VdGos1, VdSft1, VdBet1, VdVam7, VdNyv1, VdSro7, VdRab2, VdYpt52 and VdYptA. The other 15 genes showed relatively constant expression levels compared with the control during microsclerotia formation (Figure 9).

## Discussion

### Vesicle Trafficking Components in Fungi

Vesicle trafficking maintains cellular homeostasis and compartment identity in eukaryotic cells. Endocytosis and exocytosis are dependent on vesicular trafficking. Like all eukaryotes, the pathway in fungi involves distinct membrane-bound compartments interconnected by vesicular traffic. Secretory proteins and carbohydrates leave the endoplasmic reticulum in vesicles (or larger structures) bound for the Golgi apparatus [24]. Protein families called SNARE, Rab, SM and exocyst proteins mediate vesicle trafficking, especially vesicle fusion [12], [25]. In this study, we identified genes encoding SNARE, Rab, SM and exocyst proteins in *V. dahliae*. The total number of these genes was 44 and it is fewer than that of *S. cerevisiae*. We further compared the number of vesicle fusion components among other filamentous fungi, such as *V. albo-atrum*, *Fusarium spp*, *S. sclerotiorum*, *M. oryzae* and *A. oryzae* (Table 1). The compared analyses showed that the gene number of SNAREs, SM family, exocyst proteins and most Rab-GTPases in *V. dahliae* is similar to that of other filamentous fungi, suggesting the proteins involved in vesicle fusion in filamentous fungi are highly conserved. Some Rab-GTPases appeared to be highly divergent or species-specific, indicating that fungal Rab-GTPases might have specialized or general functions.

Complete and accurate annotation of genes is an essential platform for further study of evolution and gene family function. We identified 22 SNAREs, 10 Rabs, 4 SM proteins and 8 exocyst subunits from 10535 annotated genes in *V. dahliae* genome. We re-annotated these genes, by comparing alignments of orthologs from *Fusarium spp*, *S. sclerotiorum*, *M. oryzae* and *A. oryzae*, and by identifying motifs in each protein. The exon/intron structure and amino acid sequences of the ORF of these 44 genes were screened manually. We cloned and sequenced full-length cDNAs and gDNAs of genes with annotation errors, and some of these errors were corrected. For example, three introns were verified in the VdBet1 gene, and the entire amino acid sequence was corrected. Unfortunately, even using 5′ rapid amplification of cDNA ends PCR, we could not clone the 5′ sequence encoding the PX domain of VdVam7 due to incomplete genome sequence information. Anyway, general and main components involved in vesicle fusion is correctly identified and represented in our study.

### Potential Roles of Vesicle Fusion in the Secretome of *V. dahliae*

The secretome represents a catalog of the complete set of proteins secreted into the extracellular space for a given organism [60], [61]. Many secreted proteins produced by plant pathogens are pathogenicity molecules, which are secreted during host colonization and modulate host immunity [62], [63]. Klosterman et al. reported 780 potentially secreted proteins encoded in the *V. dahliae* genome, and certain secreted protein families were significantly expanded [53]. Details of the secretory pathway are limited in filamentous fungi; however, it is generally accepted that there are no major differences between yeasts and higher fungi [61]. Secreted proteins are synthesized in the classic scheme and are targeted to an organelle or the extracellular space via secretory vesicles.

Each vesicle tracking pathway can be divided into four essential steps that include vesicle budding, transport, tethering, and fusion [2]. The final fusion occur by the paring of SNAREs and their interaction with SM, Rab and tethering complexes [64]. SNAREs function as key player, so, loss of function in genes encoding SNAREs leads to deficiency of secreted proteins in plant pathogenic fungi. For example, in corn smut fungus *Ustilago maydis*, loss of a t-SNARE Yup1 resulted in a polar distribution of wall components and morphological alterations [65]; in the rice blast fungus *M. oryzae*, a *MoVam7* deletion mutant affected vacuole formation and membrane trafficking [52], and MoSec22 was found to regulate the expression of extracellular enzymes such as peroxidases and laccases [51].

The availability of the complete genomic sequence and an efficient genetic transformation system for *V. dahliae* allow the elucidation of the roles of vesicle trafficking components and, therefore, represent a challenging, but potentially valuable, basis for future functional studies. In our laboratory, the knockout VdSec22 gene significantly affected the production of melanin and extracellular substances (Sun, et al., unpublished data). It is possible that further important secreted proteins (as effectors or suppressors) will be identified among mutants of vesicle trafficking pathways.

### Biased Expression Patterns of Vesicle Fusion Components during Growth and Development of *V. dahliae*

In the present study, the expression profiles of vesicle fusion components were examined using qRT-PCR assays. The expression of all the genes was detected during vegetative growth and nearly half of the genes were differentially expressed. For example, SNARE proteins VdSso1, VdSso2, VdGos1, VdSec20, VdVti1, VdUse1, VdSft1 and VdSec22 were significantly expressed during growth and development of *V. dahliae.* Loss of SNAREs in other fungi affect fungal growth and development, i.e. MoVam7 and MoSec22 of *M. oryzae* are regulate Conidiogenesis and growth [51], [52]. In addition, SM family (VdSec1, VdSly1 and VdVps45), Rab GTPases (VdRab6 VdYpt52 and VdYptA) and exocyst proteins (VdSec8, VdSec6 and VdSec5). Differential expression of vesicle fusion components would indicate that vesicle fusion play important roles in growth and development of *V. dahliae*.

### Roles of Vesicle Fusion in Microsclerotia Formation

The formation of melanized, long-term survival structures known as microsclerotia is an important developmental event and a significant hallmark in the life cycle of *V. dahliae* [48]. Microsclerotia are the primary infectious propagules; therefore, studies on microsclerotia are a key to revealing the disease cycle and will shed light on potential avenues for the development of novel disease management strategies to combat destructive wilt diseases. Microsclerotial development has been studied at the structural and molecular level. The structures originate from hyphal swellings leading to subsequent lateral budding and formation of clusters of cells [50]. Many genes are associated with the molecular events occurring during microsclerotia formation, e.g., a hydrophobin gene VDH1 [48], [59], a mitogen-activated protein kinase VMK1 [66], a G protein β subunit [58] and a glutamic acid-rich protein VdGARP1 [67]. Little is known about the molecular pathways or gene families involved in microsclerotial development. Vesicle fusion components are highly conserved among fungi; therefore, their study may contribute to the understanding of secretory pathways proteins and their role in microsclerotia formation of *V. dahliae*.

The expression profiles of genes encoding vesicle fusion components during microsclerotia formation showed that 30 genes were significantly expressed. Interestingly, 19 genes were significantly upregulated, including 8 SNAREs. VdSec22 was highly expressed in MS initials, the loss of VdSec22 resulted in deficient of MS formation as well. Therefore, gene expression profiles of vesicle fusion would provide useful clues and help to understand molecular bases of protein secretion during darken microsclerotia and which roles of vesicle fusion machinery play in microsclerotia formation of *V. dahliae*.

### Conclusions

In this study, we identified a set of genes encoding the vesicle fusion components such as SNARE, SM, Rab and exocyst proteins in *Verticillium* and other important filamentous fungi. We analyzed their phylogeny, and performed expression profiling of 44 genes under normal vegetative growth and during microsclerotia formation. These 44 genes encode 22 SNAREs, 4 SM proteins, 10 Rab proteins and 8 exocyst components in *V. dahliae*. These sequences and expression information reported herein will be useful for further investigations of the roles of genes encoding vesicle trafficking components in the growth, development and pathogenesis of *V. dahliae*. Many of these genes are significantly differentially expressed during vegetative growth and microsclerotia formation, strongly suggesting that their participation in vesicle trafficking processes are required for microsclerotia formation, especially melanin metabolism and secreted proteins. Combined with bioinformatics analyses, the genomic structure and protein sequence of VdBet1 were revised by RT-PCR and sequencing. Although the genome sequence of *V. dahliae* has been published, functional studies on *V. dahliae* genes lag behind. Analysis of one or several gene families, rather than one gene, will be important. Based on all the results presented here, we speculate that vesicle fusion plays a critical role in microsclerotia formation. Therefore, the genome-wide identification and expression analysis of components involved in vesicle fusion should facilitate research in this gene family and give new insights toward elucidating their functions. Studies on vesicle trafficking may contribute to the understanding of secretory proteins and their roles in growth, development and pathogenesis of *V. dahliae*.

## Supporting Information

Table S1 **The complete set of SNAREs of** ***V. dahlia*** **.**
(DOC)

Table S2 **Predicted proteins of the SM family of** ***V. dahlia*** **.**
(DOC)

Table S3 **Predicted proteins of the Rab family of** ***V. dahlia*** **.**
(DOC)

Table S4 **Predicted proteins of Exocyst complex subunits of** ***V. dahlia*** **.**
(DOC)

Text S1 **The revision of VdBet1 (VDAG_06787.1) by PCR and sequencing.**
(TXT)

Dataset S1 **Complete list of the deduced amino acid sequences of SNAREs, SM proteins, Rabs and exocysts that were identified from** ***Saccharomyces cerevisiae*** **,** ***Verticillium dahlia*** **,** ***Verticillium albo*** **-** ***atrum*** **,** ***Fusarium oxysporum*** **,** ***Fusarium verticillioides*** **,** ***Fusarium graminearum*** **,** ***Sclerotinia sclerotiorum*** **,** ***Magnaporthe oryzae*** **,** ***Aspergillus oryzae***.
(XLS)
