# Supplementary material for: Genome-Wide Identification, Phylogeny and Expression Profile of Vesicle Fusion Components in Verticillium dahliae
Source: PLoS One. 2013 Jul 17;8(7):e68681. doi: 10.1371/journal.pone.0068681 (PMC3714278; doi:10.1371/journal.pone.0068681)
Supplement: Table S1 — The complete set of SNAREs of V. dahlia . (DOC) [file pone.0068681.s001.doc]

***Sup Table 1 The complete set of SNAREs of*** Verticillium dahliae

| Name | Gene ID | **Type** | **TM domain** | **Motif** | **CDS length** | **Intron**  **No.** | **Deduced protein** | | | **Scaffold information** |
| --- | --- | --- | --- | --- | --- | --- | --- | --- | --- | --- |
|  |  |  |  |  |  |  | **Length** | **MW(kDa)** | **PI** |  |
| **VdUfe1** | VDAG_10168 | Qa | - | 224-277 | 915 | 3 | 305 | 34.26 | 9.56 | 32: 151689-152961 |
| **VdSed5** | VDAG_04590 | Qa | 298-317 | 237-290 | 1083 | 1 | 261 | 35.44 | 9.09 | 8: 86793-87931 |
| **VdTlg2** | VDAG_04950 | Qa | 324-341 | 260-313 | 1433 | 4 | 320 | 40.21 | 5.68 | 8: 1334607-1336324 |
| **VdSso1** | VDAG_00278 | Qa | 313-335 | 248-301 | 1591 | 2 | 333 | 45.17 | 6.10 | 1: 910862-912591 |
| **VdSso2** | VDAG_06050 | Qa | 303-325 | 239-292 | 1655 | 3 | 409 | 37.51 | 5.51 | 13: 276362-278265 |
| **VdPep12** | VDAG_07498 | Qa | 244-263 | 180-233 | 795 | 1 | 265 | 29.88 | 4.93 | 17: 75589-76449 |
| **VdSec20** | VDAG_04124 | Qb | - | 195-248 | 1209 | 1 | 403 | 44.49 | 4.67 | 7: 133165-134443 |
| **VdBos1** | VDAG_08270 | Qb | 254-273 | 193-246 | 828 | 1 | 276 | 30.62 | 9.66 | 19: 676789-677685 |
| **VdGos1** | VDAG_04359 | Qb | 207-224 | 147-200 | 226 | 2 | 678 | 25.32 | 8.95 | 7: 884214-885033 |
| **VdVti1** | VDAG_01975 | Qb | 202-224 | 145-198 | 116 | 1 | 229 | 25.33 | 8.07 | 3: 1112687-1113935 |
| **VdSec9** | VDAG_04327 | Qbc | - | 268-321 | 1443 | 2 | 481 | 51.06 | 6.22 | 7: 796955-798537 |
| **VdUse1** | VDAG_05740 | Qc | 324-346 | 263-316 | 1062 | 2 | 354 | 38.60 | 5.38 | 11: 320015-321194 |
| **VdSft1** | VDAG_05532 | Qc | - | 14-58 | 213 | 0 | 71 | 7.92 | 7.90 | 10: 814723-814935 |
| **VdBet1** | VDAG_06787 | Qc | 147-167 | 89-142 | 513 | 3 | 171 | 18.38 | 9.47 | 14: 621423-622077 |
| **VdTlg1** | VDAG_01350 | Qc | 220-242 | 160-213 | 816 | 2 | 272 | 29.93 | 4.75 | 2: 1610647-1611881 |
| **VdSyn8** | VDAG_01236 | Qc | 269-286 | 207-260 | 864 | 1 | 288 | 31.42 | 5.36 | 2: 1278876-1279825 |
| **VdVam7** | VDAG_03579 | Qc | - | 76-125 | 387 | 1 | 129 | 13.95 | 8.04 | 6: 68929-69463 |
| **VdSec22** | VDAG_08386 | R | 193-215 | 137-190 | 654 | 2 | 218 | 25.29 | 8.88 | 20: 262475-263250 |
| **VdYkt6** | VDAG_08948 | R | - | 138-191 | 693 | 6 | 198 | 22.42 | 7.05 | 24: 233918-235083 |
| **VdSnc1** | VDAG_02648 | R | 96-118 | 33-86 | 363 | 2 | 121 | 13.21 | 9.30 | 4: 952957-953837 |
| **VdNyv1** | VDAG_10121 | R | 216-235 | 153-206 | 817 | 1 | 238 | 26.08 | 8.55 | 32: 17368-18237 |
| **Vd** **Sro7** | VDAG_10481.1 | R | - | 509-895 | 3099 | 3 | 969 | 335.42 | 5.28 | 41: 17412-20510 |
