# Supplementary material for: Genome-Wide Identification, Phylogeny and Expression Profile of Vesicle Fusion Components in Verticillium dahliae
Source: PLoS One. 2013 Jul 17;8(7):e68681. doi: 10.1371/journal.pone.0068681 (PMC3714278; doi:10.1371/journal.pone.0068681)
Supplement: Table S2 — Predicted proteins of the SM family of V. dahlia . (DOC) [file pone.0068681.s002.doc]

**sup Table 2 Predicted proteins of the Sec1 family of *Verticillium dahliae***

| Name | Gene ID | **TM**  **domain** | Motif | **CDS length** | **Intron**  **No.** | **Deduced protein** | | | **Scaffold information** |
| --- | --- | --- | --- | --- | --- | --- | --- | --- | --- |
|  |  |  |  |  |  | **Length** | **MW** | **PI** |  |
| **VdSly1** | VDAG_02581 | - | 53-655 | 2001 | 6 | 667 | 72.84 | 5.08 | 4: 743893-746342 |
| **VdVps45** | VDAG_06294 | - | 29-566 | 2303 | 2 | 585 | 65.29 | 6.24 | 12: 25935-28323 |
| **VdSec1** | VDAG_10115 | - | 32-461 | 1842 | 2 | 614 | 68.96 | 8.78 | 32: 3394-5991 |
| **VdVps33** | VDAG_04476 | 588-610 | 33-635 | 1965 | 3 | 655 | 73.08 | 6.47 | 7: 1217273-1219478 |
