# Supplementary material for: Genome-Wide Identification, Phylogeny and Expression Profile of Vesicle Fusion Components in Verticillium dahliae
Source: PLoS One. 2013 Jul 17;8(7):e68681. doi: 10.1371/journal.pone.0068681 (PMC3714278; doi:10.1371/journal.pone.0068681)
Supplement: Table S3 — Predicted proteins of the Rab family of V. dahlia . (DOC) [file pone.0068681.s003.doc]

**sup Table 3 Predicted proteins of the Rab family of *Verticillium dahliae***

| Name | Gene ID | **Motif** | **CDS length** | **Intron**  **No.** | **Deduced protein** | | | **Scaffold information** |
| --- | --- | --- | --- | --- | --- | --- | --- | --- |
|  |  |  |  |  | **Length** | **MW** | **PI** |  |
| **VdRad8** | VDAG_01942 | 12-172 | 865 | 2 | 209 | 22.70 | 6.60 | 3: 1029186-103027 |
| **VdRad7** | VDAG_01437 | 10-175 | 1091 | 6 | 206 | 22.91 | 4.86 | 2: 1885937-1887394 |
| **VdYpt1** | VDAG_07163 | 10-170 | 775 | 4 | 203 | 22.46 | 5.31 | 15: 874401-875602 |
| **VdRad11** | VDAG_00886 | 11-171 | 989 | 4 | 213 | 23.25 | 5.46 | 2: 262059-263519 |
| **VdRad2** | VDAG_03931 | 32-243 | 840 | 0 | 280 | 35.28 | 9.25 | 6: 1255950-1256789 |
| **VdRad4** | VDAG_06179 | 17-206 | 1055 | 3 | 335 | 25.82 | 4.82 | 13: 719837-721104 |
| **VdYpt52** | VDAG_05807 | 7-138 | 744 | 2 | 248 | 37.62 | 6.72 | 11: 529976-530892 |
| **VdYptA** | VDAG_08951 | 55-208 | 1053 | 1 | 351 | 26.90 | 9.69 | 24: 243602-244748 |
| **VdYpt53** | VDAG_06883 | 135-179 | 825 | 3 | 244 | 22.00 | 9.23 | 14: 920027-921040 |
| **VdRad6** | VDAG_10156 | 11-52; 112-237 | 591 | 3 | 197 | 29.62 | 6.96 | 32: 106592-107800 |
