# Supplementary material for: Genome-Wide Identification, Phylogeny and Expression Profile of Vesicle Fusion Components in Verticillium dahliae
Source: PLoS One. 2013 Jul 17;8(7):e68681. doi: 10.1371/journal.pone.0068681 (PMC3714278; doi:10.1371/journal.pone.0068681)
Supplement: Table S4 — Predicted proteins of Exocyst complex subunits of V. dahlia . (DOC) [file pone.0068681.s004.doc]

**sup Table 4 Predicted proteins of Exocyst complex subunits of *Verticillium dahliae***

| Name | Gene ID | Motif | **CDS length** | **Intron**  **No.** | **Deduced protein** | | | **Scaffold information** |
| --- | --- | --- | --- | --- | --- | --- | --- | --- |
|  |  |  |  |  | **Length** | **MW** | **PI** |  |
| **VdExo84** | VDAG_00536 | 141-224 | 2064 | 3 | 688 | 77.55 | 9.30 | 1: 1764814-1767049 |
| **VdSec10** | VDAG_03094 | 109-844 | 2553 | 1 | 851 | 94.44 | 5.53 | 5: 288653-291267 |
| **VdSec8** | VDAG_08435 | 84 -223 | 3258 | 1 | 1086 | 121.43 | 5.45 | 20: 433190-43651 |
| **VdExo70** | VDAG_09051 | 247-628 | 2293 | 1 | 634 | 69.99 | 9.01 | 24: 542540-544898 |
| **VdSec6** | VDAG_04051 | 175-750 | 2256 | 0 | 752 | 87.15 | 4.98 | 6: 1641657-1643912 |
| **VdSec15** | VDAG_06597 | 449-737 | 2608 | 9 | 762 | 86.66 | 4.94 | 14: 14424-17628 |
| **VdSec3** | VDAG_05501 | 663-1368 | 4167 | 5 | 1389 | 153.43 | 7.95 | 10: 677757-682197 |
| **VdSec5** | VDAG_00456 | 115-181 | 3180 | 2 | 1060 | 116.98 | 6.83 | 1: 1521801-1525104 |
